# Comparative Analysis of Heavy Metal Leaching from Metallurgical Slag Using Different Types of Leaching Agents: A Kinetic Models Approach

**DOI:** 10.3390/molecules31193386

**Published:** 2026-09-23

**Authors:** Krzysztof Barbusiński, Milena Nocoń, Krzysztof Piotrowski, Krzysztof Loska, Irena Korus

**Affiliations:** 1 Silesian University of Technology, Faculty of Energy and Environmental Engineering, Department of Water and Wastewater Engineering, Konarskiego 18, 44-100 Gliwice, Poland; krzysztof.barbusinski@polsl.pl (K.B.); krzysztof.loska@polsl.pl (K.L.); irena.korus@polsl.pl (I.K.); 2 Silesian University of Technology, Faculty of Chemistry, Department of Chemical Engineering and Process Design, ks. M. Strzody 7, 44-100 Gliwice, Poland; krzysztof.piotrowski@polsl.pl

**Keywords:** metallurgical slags, solid–liquid extraction, leaching, heavy metals, kinetic modeling

## Abstract

In accordance with the Circular Economy, the metallurgical solid wastes are considered secondary resources of many heavy metals, such as lead, zinc, iron, cadmium, thallium, and others. To separate valuable and toxic metals, numerous processes, among them solid–liquid extraction, have been studied in recent years. In line with this trend, the objective of this study was to investigate the possibility of leaching selected heavy metals (Pb, Zn, Cu, and Fe) from slag generated by the zinc and lead metallurgical industry. The effect of the leaching agent type (HCl, NaOH, and Na_2_NTA) and concentration, pH, solid-to-liquid (S/L) ratio, and interphase contact time on process efficiency was evaluated experimentally. The slag was comprehensively characterized using atomic absorption spectrometry (AAS), scanning electron microscopy coupled with energy-dispersive X-ray spectroscopy (SEM-EDS), X-ray diffraction (XRD), and BCR (*Community Bureau of Reference*) sequential extraction to determine the total metal concentration, element and phase composition, as well as chemical speciation of the investigated metals. To determine the kinetic mechanisms the Weber–Morris and shrinking-core models were used. The efficiency of metal separation from slag strongly depended on the process parameters. HCl was highly effective for simultaneous metal leaching, particularly Pb, Zn, and Cu (up to over 80%), while NaOH and Na_2_NTA allowed for the selective separation of Pb with leaching efficiencies of up to 80% and 90%, respectively. Sequential BCR analysis confirmed the different forms of metal occurrence, with predominating highly mobile Pb fractions and stable, residual Cu and Fe fractions. Comparative analyses using SEM and sequential BCR extraction revealed significant changes in slag morphology and metal speciation following leaching with the HCl solution. The Weber and Morris model indicated the kinetic contribution of the intraparticle diffusion within the slag’s porous structure, especially under HCl-based leaching. An analysis using shrinking-core models revealed different predominant mechanisms for specific metal–leaching agent systems.

## 1. Introduction

Heavy metals present in metallurgical slags pose a significant environmental and technological challenge due to their toxicity, persistence, and potential release under changing environmental conditions [1]. However, these materials represent valuable secondary resources of metals, which makes their selective recovery an important aspect of modern hydrometallurgical processes and Circular Economy standards [2]. Leaching, also referred to as solid–liquid extraction, is widely applied as an effective method for hydrometallurgical separation of metals from complex solid matrices [3], especially from primary ores, metallurgical slags, fly ashes, mine tailings, and other industrial residues [2,4]. The process is based on the transfer of soluble metal species from a solid phase into a liquid medium through dissolution (sometimes with auxiliary chemical reactions) occurring at the solid–liquid interface. Depending on the chemical composition and mineralogical structure of the material, metals may be identified in different forms, including oxides, sulfides, silicates, spinels, berthollides, or some amorphous phases, differing considerably in their susceptibility to dissolution. Consequently, the effectiveness of leaching is closely related to the compliance and chemical stability of metal-bearing phases within the solids [5,6,7].

The efficiency and selectivity of heavy metal leaching from metallurgical slags strongly depend on the chemical nature of the leaching agent, which determines the dissolution and/or complexation process governing the transfer of metals from the solid to the liquid phase [8]. Depending on the target metals and the mineralogical composition of the slag, inorganic acids, alkaline solutions, and complexing agents have been successfully employed for metal leaching from metallurgical and other industrial wastes [8,9,10,11]. Acidic leaching agents are often used in studies on the separation of heavy metals from metallurgical waste [9,11,12,13]. Acidic leaching promotes the dissolution of acid-soluble and acid-reactive mineral phases by proton reaction with solid matrix, thereby facilitating the release of associated metal ions into solution [14]. Among inorganic acids, HCl is a commonly applied leaching agent because of its strong acidity and its ability to promote the dissolution of a wide range of metal-bearing phases. In addition, chloride ions may participate in the complexation of dissolved metal species, which can further influence their behavior in the leaching solution [15,16]. Alkaline leaching provides a chemically different environment and may offer greater selectivity toward metals whose hydroxide or oxide chemistry is strongly pH-dependent, particularly amphoteric elements such as Fe, Cu, Cd, Co, Ni, etc. [17]. NaOH is therefore frequently used as a representative strong alkaline leaching agent for the selective dissolution of metals from complex industrial residues. In contrast, complexing agents can enhance metal dissolution through the formation of soluble metal–ligand complexes, thereby shifting the dissolution equilibrium and facilitating the transfer of metal ions into solution [18,19]. Na_2_NTA (disodium nitrilotriacetate) was selected in the present study as a representative complexing agent because NTA is capable of coordinating a range of metal ions and has been considered a more environmentally favorable alternative to less biodegradable complexing agents such as EDTA [20,21]. The ability of NTA to bind dissolved metal ions is also strongly dependent on its protonation state and therefore on solution pH, making pH an important parameter in NTA system leaching. The performance of the leaching process is influenced not only by the chemical nature of the leaching medium and the metal-bearing phases, but also by process parameters such as concentration of the leaching agent, pH (especially for complexing agents), solid-to-liquid (S/L) ratio, and interphase contact time [9,15,16,22,23]. These variables affect both the extent of metal release to solution and the process selectivity toward target elements. Consequently, kinetic analysis provides information complementary to final leaching efficiency by helping to identify the stages that may control the overall rate of metal release. Previous studies on the leaching of metals from industrial solid wastes have demonstrated that different kinetic models, including shrinking-core and diffusion-based approaches, can provide insight into changes in the apparent rate-controlling regime under different leaching conditions [24].

The modified BCR (*Community Bureau of Reference*) sequential extraction procedure is commonly employed to determine the distribution and potential mobility of metals in complex solid matrices [25,26,27]. The method fractionates metals into operationally defined phases, characterized by different binding strengths and environmental stability [28]. Four fractions are typically distinguished: (F1) the acid-soluble/exchangeable fraction (the most mobile fraction), (F2) bound to iron and manganese oxides/reducible fraction (mobile fraction), (F3) fraction associated with organic matter and sulfides/the oxidizable fraction (potentially mobile fraction), (F4) the residual fraction, incorporated within the crystal lattice of primary and secondary minerals (non-mobile fraction). The modified BCR sequential extraction procedure is one of the most commonly used approaches for assessing metal speciation and potential mobility in metallurgical slags [29]. The obtained results enable the assessment of metal mobility, potential environmental risk, and the stability of metal-bearing phases under changing physicochemical conditions, which is particularly important for evaluation the management, utilization, or disposal of metallurgical residues.

Although a range of leaching agents and characterization methods have been applied to metallurgical slags, comparative studies involving leaching systems with substantially different chemical properties are less common, particularly when several key process parameters are systematically evaluated for the same slag matrix. Such an approach is important for complex zinc–lead slags, where the response of individual metals may differ considerably depending on their chemical form and the chemistry of the leaching medium. Using the same slag matrix for different leaching system also enables a more direct comparison of the effects of leaching chemistry, without the additional influence of differences in the initial composition and mineralogy that may complicate comparison among studies performed on different slag types.

The aim of this study was to investigate the behavior of selected heavy metals (Pb, Zn, Cu, and Fe) during co-leaching from actual metallurgical slag waste under various extraction conditions. Three different types of leaching agents, representing acidic (HCl), alkaline (NaOH), and complexing (Na_2_NTA) systems, were evaluated to assess their effectiveness and selectivity toward metal leaching. Particular attention was paid to the influence of the leaching agent type and concentration, as well as selected operational parameters, including pH (for Na_2_NTA), solid-to-liquid (S/L) ratio, and interphase contact time on the leaching efficiency of these metals. The effects of these parameters were evaluated comparatively for the same slag matrix, allowing the responses of Pb, Zn, Cu, and Fe to chemically different leaching environments to be directly assessed. In addition, the chemical speciation of the investigated metals was evaluated by the modified BCR sequential extraction procedure for both the untreated slag and the solid residues obtained after a controlled laboratory-scale leaching process, allowing the changes in metal distribution and mobility to be identified. Morphological changes resulting from acid leaching were further investigated using scanning electron microscopy (SEM) by comparing the untreated slag with the residue after HCl treatment. To evaluate the kinetic mechanisms involved in the leaching process, the experimental data were analyzed using the Weber–Morris model and the shrinking-core models. This kinetic analysis was used to examine differences in the rate-controlling behavior of individual metals and leaching systems rather that evaluation the process solely on the basis of final leaching efficiency. Overall, this study combines comparative evaluation of chemically distinct leaching systems, systematic investigation of relevant process parameters, metal-specific kinetic analysis, and post-leaching speciation to provide a more comprehensive understanding of metal leaching behavior in the investigated zinc–lead metallurgical slag.

## 2. Calculations

### 2.1. Kinetic Model Analysis

In the case of solids (like slag) with a natural internal porous structure, or where the porous structure results directly from the leaching process of the soluble fraction from an inert matrix (and is therefore dependent on the process parameters), resistance to intraparticle diffusion can significantly influence the process rate [30]. This aspect is, however, crucial in industrial-scale technological applications, determining the required interphase contact time to achieve the desired leaching effect. Complex solid leaching processes involve the simultaneous occurrence of a number of diffusion phenomena, with different rates resulting from the transport of leached components under different conditions. These include external diffusion transport of leaching and leached components through the liquid boundary layer and, above all, internal diffusion processes in porous structures with varying pore sizes (macropores, mesopores, micropores). The Bangham, Crank, and Weber–Morris models [31], among others, are used to analyze these processes and identify the dominant consecutive internal diffusion mechanisms [32]. In addition, for external diffusion, the Spahn–Schlunder or Boyd models are used [31].

### 2.2. Kinetic Modeling: Weber–Morris Model

The Weber–Morris model is most often used to analyze kinetic relations in solid-fluid processes. This model is based on the assumption that intraparticle diffusion plays a decisive role in mass transport processes, constituting a rate-limiting step for the entire solvent leaching. It allows for the determination of time intervals for individual diffusion (or other slowing down) mechanisms and the identification of the “critical time points” of mass transfer mechanism changes, which may be important for technological decisions related to the selection of optimal conditions for this process. It should be noted, that the use of pseudo-first-order or pseudo-second-order kinetic models alone does not allow for the identification of individual kinetic stages of the heterogeneous solvent leaching process [33]. Therefore, the Weber–Morris intraparticle diffusion model can be used for the initial assessment of potential rate-controlling processes in post-process slag leaching.

The equation related to the Weber–Morris model can be written as follows:

(1) $$X=K_{id}t^{0.5}+C$$

where *X*—leaching efficiency; *t*—time, min; *K*_id_—intraparticle diffusion rate constant, min^−0.5^; *C*—the Weber–Morris model constant.

The Weber–Morris model (2) [34,35,36,37,38,39] uses normalized leaching efficiency values *X* (in the range of 0–1). Considering the theoretical basis of the model, the linearity of the relationship *X = f*(*t*^0.5^) for all experimental leaching data may indicate a potential contribution of intraparticle diffusion is a controlling phenomenon in the leaching; however, it is not the only phenomenon observed in this process system [40,41]. Only when relationship (2) is not only linear but also passes through the origin (thus *C* = 0), can it be approximately assumed that the only resistance to mass transfer in the leaching process results from complex intraparticle (pore) diffusion phenomena [34,40]. In such a situation, the indirectly identified thickness of the external diffusion layer in the surrounding liquid is negligible and can be omitted in the analysis of diffusional resistances in the series connection. However, for larger values of *C*, this resistance must be taken into account.

The subsequent linear segments often identified for experimental data in the coordinate system *X vs. t*^0.5^ can be interpreted as successive stages of the mass transfer process, associated with increasingly higher resistances, as evidenced by the systematic reduction in the *K*_id_ coefficient value for subsequent stages. Here, the potential change in the pore diameter distribution of the porous structure (natural or/and generated directly within the inert matrix during the leaching process) plays a decisive role—macropores, mesopores, and micropores as a system for distributing the leaching agent into the interior of the leached material [38,41,42,43,44,45,46,47]. This may indirectly confirm the existence (or in situ self-formation) of several segments in the pore size distribution, which can have a pronounced effect on the kinetics of mass transfer in the leaching process. It should be emphasized that, at the same time, the extension of the diffusion path to increasingly distant locations within the porous structure of the solid will play an increasingly important role in the subsequent stages of the process. This extension, together with the systematic reduction of the pore diameter, may influence the slowing down of the leaching process through some synergy effects, which may be of great technological importance (the need for mechanical preprocessing of raw solids—with the indication of the optimal final grain size, economical time of the solid–leaching agent interphase contact for some assumed economic efficiency of leaching, etc.).

### 2.3. Kinetic Analysis: Shrinking-Core Models

Chemical reactions or some other forms of local (interface) non-catalytic physicochemical interactions between solid and liquid phases can be generally described using two types of theoretical models developed for ideal process conditions. These are the progressive conversion model and the unreacted shrinking-core model—of constant particle size and of shrinking particle size variants [48,49].

Kinetic analysis of the leaching process based on shrinking-core models involves identifying the number and type of potential physicochemical interactions (reaction, dissolution, etc.) on the surface of the unreacted particle fraction involving the leaching fluid action. Under such defined conditions in a heterogeneous system, the unreacted fraction systematically decreases in volume as the leaching process progresses, while the thickness of the layer of emerging porous material (inert residue) systematically increases, thus continuously increasing the diffusion path length (including back diffusion of mobilized separated components).

Shrinking-core models can be applied to analyze the kinetic data to assess the potential contribution of different physicochemical mechanism(s) and their apparent rates during metal leaching from, e.g., the post-process slag. These models, originally developed for gas–solid combustion systems [50], are often used due to their similar mechanisms to determine and analyze the mechanism of non-catalytic heterogeneous reactions/processes involving liquid and solid phases [51,52,53,54,55,56,57,58,59,60] or to interpret data from isothermal thermogravimetric curves [61,62].

The rate of the leaching process is directly dependent on the rate of the slowest of the three stages of the leaching sub-processes connected in series:

- Diffusion through the liquid film surrounding each solid particle;
- Diffusion through the growing product layer, ash, or porous structure represented by the inert fraction of the leached solid;
- Chemical reaction or any other physicochemical interaction with the unreacted core surface.

According to the theoretical foundations of these models, the rate of the physicochemical leaching process can be controlled by one or a combination of several mechanisms. The mechanisms and the corresponding kinetic rate equations in integrated form include [63,64,65,66,67,68,69,70,71]:

- A chemical reaction at the interface between the leaching agent and the solid reactant (the Spenser–Topley–Kewan model)

(2) $$1-{\left(1-X\right)}^{\frac{1}{3}}=k_{1}t$$

- The diffusion of reacting components or products (back-diffusion) through the newly formed product(s) layer or ash residue

(3) $$1-3{\left(1-X\right)}^{\frac{2}{3}}+2\left(1-X\right)=k_{2}t$$

- The diffusion of components through the near-surface layer of the liquid film

(4) $$X=k_{3}t$$

- Directly related, sequential or parallel processes of interface transfer and diffusion across the product ash (e.g., porous structure) layer

(5) $$\frac{1}{3}Ln\left(1-X\right)+\left[{\left(1-X\right)}^{-1/3}-1\right]=k_{4}t$$

where *X*—leaching efficiency (in the range of 0–1); *t*—time, min; *k*_1_, *k*_2_, *k*_3_, and *k*_4_—the apparent rate constant in Equations (2)–(5), min^−1^.

Shrinking-core models were used to describe the kinetics and identify the non-catalytic heterogeneous process mechanisms by many authors. Kassab et al. [72] applied these models to the hydrometallurgy leaching of REEs, Zn, and Ni using sulfuric acid and ammonium sulfate. Ismael et al. [63] used them to study the leaching kinetics and mechanism of Ti from a rutile concentrate using sulfuric acid solution as the leaching agent.

## 3. Results and Discussion

### 3.1. Sample Analysis

The slag sample was collected from the Short Rotary Kiln at the Zinc Smelter in the form of hard, irregularly shaped rock of a dark gray color (Figure 1a). A preliminary analysis of the sample’s external structure revealed significant variation in surface texture, shape, and metallic inclusions. The processing of the material (crushing, drying, grinding) resulted in an unfractionated slag sample (Figure 1b). Furthermore, it showed low moisture content (0.04 ± 0.009% _mass_) determined at 105 °C and relatively high hardness during further processing (grinding in a ball mill).

A qualitative and quantitative analysis of the metals present in the material, using atomic absorption spectrometry (AAS) (Figure 2a), revealed that iron was the predominant quantified metal, with a concentration of 286.1 g/kg, followed by Zn (122.8 g/kg), Pb (61.8 g/kg), Ca (29.7 g/kg), and Cu (23.1 g/kg). The remaining quantified metals, including Mn, K, Na, Cr, and Ni, were present at substantially lower concentrations. When considering only the metals quantified by AAS, Fe accounted for 54.1% of their total measured concentration, followed by Zn (23.2%), Pb (11.7%), Ca (5.6%), and Cu (4.4%). These percentages refer to the relative contribution of each quantified metal to the total concentration of metals determined by AAS and not to the total mass of the slag. The predominance of iron observed in the investigated slag is consistent with the chemical composition commonly reported for zinc–lead metallurgical slags, in which Fe-rich oxide phases constitute the major component of the slag matrix. The relatively high contents of Zn and Pb indicate incomplete recovery of valuable metals during the smelting process, while their concentrations remain within the range reported for pyro-metallurgical Zn–Pb slags. Similar chemical characteristics have been described by Nowińska and Adamczyk [6], Król et al. [23], as well as Piatak et al. [1], who reported that the composition and appearance of zinc slags are strongly influenced by the feed material and technological parameters of the metallurgical process.

The XRD phase analysis revealed a significant diversity of crystalline mineral phases present in the slag sample (Figure 2b). The types of chemical compounds present in the material might depend heavily on the ore concentrate used in the processing, the conditions under which the processing was carried out, as well as the refining process conditions. The material contains simple metal oxides and sulfides, as well as spinels and various types of silicates, which are often found in similar types of materials [6,13,23,73,74]. In addition, Pb was identified among the crystalline phases, together with several non-stoichiometric compounds. Metallic inclusions of individual elements may also occur in metallurgical slags [75]; however, their unambiguous identification was beyond the scope of the present qualitative XRD analysis. Due to the presence of a significant amorphous phase, reliable quantitative phase analysis could not be performed. Consequently, the XRD investigation was limited to qualitative identification of the crystalline phases. Similar limitations have been reported for metallurgical slags by Piatak et al. [1], who emphasized the difficulties associated with quantifying crystalline and amorphous constituents using conventional XRD.

The SEM observations revealed that the investigated material showed heterogeneous contrast in the backscattered electron (BSE) image. The contrast observed in the SEM-BSE image is governed by the average atomic number of the constituent phases. Consequently, phases enriched in heavy elements (e.g., Pb, Cu, Sn, and Zn) appear brighter, whereas silicate-rich phases containing predominantly Si, O, Ca, Na, and Al exhibit darker gray contrast. The exemplary SEM-BSE image of the tested sample (Figure 3) revealed three regions with distinct gray levels, indicating differences in chemical composition. The medium-gray area (Point 1) is dominated by iron (72.3% _mass_) and contains a significant amount of zinc (15.1% _mass_), together with minor tin (1.4% _mass_) and lead (0.5% _mass_). The relatively high iron content combined with oxygen (9.2% _mass_) may be attributed to Fe-rich oxide phases containing minor amounts of Zn and Sn. The dark-gray region (Point 2) exhibits a more complex chemical composition. It contains elevated concentrations of zinc (25.1% _mass_), oxygen (24.1% _mass_), silicon (16.8% _mass_), and iron (15.7% _mass_), together with sodium, calcium, and minor amounts of tin and sulfur. This composition indicates a heterogeneous silicate–oxide phase enriched in zinc, likely formed during the cooling and solidification of the slag. The brightest region (Point 3) is characterized by very high concentrations of copper (52.7% _mass_) and tin (27.9% _mass_), accompanied by lead (7.7% _mass_) and only a small amount of iron (1.7% _mass_). Such a composition suggests the occurrence of a metallic Cu-rich phase enriched in Sn and Pb. The high average atomic number of these elements explains the bright contrast observed in the BSE image. The heterogeneous distribution of bright metallic inclusions and darker silicate-rich domains observed in the BSE images reflects the complex microstructure of the investigated slag. Similar microstructural heterogeneity and elemental distribution have been reported for Zn–Pb and Cu metallurgical slags by Warchulski and Szopa [76], Ettler et al. [77], and Nowińska and Kokowska-Pawłowska [5].

### 3.2. Concentration, pH, and Type of Leaching Agents

The leaching efficiency determined for four heavy metals—Pb, Zn, Cu, and Fe—as a function of the type and concentration of individual leaching agents is shown in Figure 4a–c. In addition, the effect of the varying pH of the complexing leaching agent solution (Na_2_NTA) on the leaching efficiency of these metals is shown in Figure 4d.

The slag exhibits a high susceptibility to leaching in HCl solution over a wide range of concentrations, as indicated by the leaching efficiency data presented in Figure 4a. A noticeable leaching efficiency for all the metals studied (above 50%) was observed even at a relatively low acid concentration (0.5 mol/dm^3^), while the maximum leaching efficiency for Pb, Cu and Fe was achieved using a 1.5 mol/dm^3^ HCl solution. The high leaching efficiencies obtained with hydrochloric acid solution indicate that a significant portion of the investigated metals occurs in acid-soluble phases, including oxides and other readily reactive mineral forms. Moreover, the extremely low pH of the leaching system promotes the dissolution of both the primary metal-bearing phases and the secondary reaction products formed during leaching, thereby preventing their precipitation and facilitating the continuous release of metal ions into the solution. The high leaching efficiency of hydrochloric acid observed in the present study is consistent with other investigations demonstrating that HCl is an effective lixiviant for extracting heavy metals from a wide range of metallurgical wastes [15,78,79]. However, the optimum acid concentration reported in the literature varies from dilute solutions to concentrated hydrochloric acid, depending primarily on the mineralogical composition, metal speciation, and physicochemical properties of the investigated material as well as the intended recovery process [15,16]. Consequently, the optimum HCl concentration is generally selected according to the characteristics of the specific waste and the objectives of the leaching process.

In contrast, alkaline leaching using NaOH solution (Figure 4b) and complexing leaching with Na_2_NTA solution (Figure 4c) exhibited distinctly different behaviors. In both systems, Pb was almost completely removed even at the lowest reagent concentrations, whereas the leaching efficiencies of the remaining metals were substantially lower. Copper leaching reached approximately 20% (being slightly higher in NaOH than in Na_2_NTA solution), while zinc leaching did not exceed 5%. Iron remained practically insoluble (and nonleachable) under both alkaline and complexing conditions. The high selectivity toward Pb can be attributed primarily to its occurrence in the most mobile fraction, which is consistent with the sequential extraction results presented in Section 3.6.1. Similar selective leaching of Pb using NTA solution has been reported by Giannis et al. [80], who also demonstrated that the leaching efficiency of heavy metals depends strongly on solution pH and metal speciation. In contrast, the poor dissolution of Fe suggests that it is predominantly incorporated into stable iron oxide or spinel-like phases that are resistant to alkaline attack. Similarly, the limited leaching of Zn and Cu indicates that these metals might be mainly bound to mineral phases that are structurally incorporated into the slag matrix, making them considerably less susceptible to dissolution under alkaline conditions. These findings demonstrate that NaOH and Na_2_NTA are highly selective leaching agents for Pb removal, while hydrochloric acid is considerably more effective when the simultaneous extraction of several metals is required under the applied laboratory-scale conditions.

The effect of Na_2_NTA solution pH on metal leaching efficiency is presented in Figure 4d. The leaching efficiency of Pb increased with increasing pH, rising from approximately 45% at pH 2 to about 65% at pH 8, after which no further improvement was observed. This trend is consistent with the pH-dependent speciation of NTA. Increasing the solution pH shifts the acid-base equilibrium of NTA toward more highly deprotonated ligand species. These species have a greater ability to coordinate dissolved metal ions, which can enhance metal complexation and facilitate their transfer into the solution [80,81]. In contrast, Zn exhibited the highest leaching efficiency at pH 2 (approximately 20%), followed by a gradual decrease to about 3% at pH 12. The enhanced leaching of Zn under acidic conditions may result from the combined action of proton-assisted dissolution and complex formation, whereas, at higher pH values, zinc is more strongly retained within the slag matrix or occurs in less soluble mineral phases. Copper leaching remained nearly constant at approximately 5% across the entire pH range, indicating that changes in pH had little influence on its leaching. Iron was practically insoluble under all investigated conditions, suggesting that it is predominantly associated with highly stable iron-bearing phases that are immobile under both types of test conditions (changing the concentration and the pH). The different responses of Pb, Zn, Cu, and Fe to changes in solution pH indicate that the effect of pH is strongly metal-specific and results from the combined influence of ligand speciation, metal complexation, and the dissolution behavior of individual mineral phases. Similar observations were reported by Naghipour et al. [82], who demonstrated that the optimum pH for NTA-assisted extraction varied among metals.

### 3.3. Solid-to-Liquid (S/L) Ratio

The effect of the solid-to-liquid (S/L) ratio on metal leaching efficiency using HCl, NaOH, and Na_2_NTA solutions is presented in Figure 5a, Figure 5b and Figure 5c, respectively.

Regardless of the leaching agent applied, lead exhibited a similar response to changes in the S/L ratio. At the lowest S/L (1/200), Pb extraction reached approximately 90%, 88%, and 70% for HCl, NaOH, and Na_2_NTA, respectively. Increasing the S/L ratio up to 1/100 resulted in the highest leaching efficiencies, exceeding 95% for HCl, 90% for NaOH, and 80% for Na_2_NTA. A further increase in the S/L value (1/50) caused a pronounced decrease in Pb extraction, with the leaching efficiencies decreasing to approximately 55%, 60%, and 45%, respectively. The observed decline at the highest S/L ratio is most likely associated with the limited availability of the leaching (re)agent per unit mass of slag, resulting in insufficient dissolution capacity and a lower extent of Pb release into the solution.

The leaching behavior of Zn was strongly dependent on the type of leaching agent employed. In the HCl system, zinc extraction followed a trend similar to that observed for Pb, with the maximum leaching efficiency (approximately 90%) obtained at an S/L ratio of 1/100. At S/L ratios of 1/200 and 1/50, the leaching efficiencies were considerably lower and remained at a comparable level of approximately 70%. In contrast, Zn dissolution in NaOH solution was limited throughout the investigated range, with leaching efficiencies below 10%. A slight decrease in Zn leaching was observed with increasing S/L ratio; however, the differences were relatively small. The lowest Zn recovery was obtained using Na_2_NTA solution. The highest leaching efficiency, approximately 10%, was achieved at an S/L ratio of 1/200, followed by a noticeable decrease at 1/100 and a further reduction to only a few percent at an S/L ratio of 1/50.

Copper and iron exhibited distinctly different leaching behavior depending on the reagent used. In the HCl solution, Cu extraction was high for all investigated S/L ratios, reaching approximately 80%, 90%, and 82% for S/L ratios of 1/200, 1/100, and 1/50, respectively. A slight improvement in Cu leaching was observed at an S/L ratio of 1/100. In contrast, the leaching efficiency of Cu remained nearly constant over the entire S/L range in both NaOH and Na_2_NTA systems, indicating that controlled changes in the solid loading can provide only a limited influence on its dissolution under alkaline or complexing conditions. A different trend was observed for Fe. Iron was practically insoluble in both NaOH and Na_2_NTA environments, regardless of the S/L ratio, confirming its strong association with chemically stable iron-bearing phases. In hydrochloric acid solution, however, Fe was readily leached, with extraction efficiencies of approximately 80%, 72%, and 80% for S/L ratios of 1/200, 1/100, and 1/50, respectively. Unlike Pb and Zn, iron did not exhibit a clear dependence on the S/L ratio, suggesting that its dissolution was primarily controlled by the action of the acidic leaching solution on Fe-rich mineral phases rather than by reagent availability within the investigated range of process parameters.

Overall, an S/L ratio of 1/100 provided the most favorable operating conditions for the investigated slag, particularly with respect to the simultaneous recovery of Pb, Zn, and Cu. The importance of the solid-to-liquid ratio has been highlighted in other hydrometallurgical studies. For example, Trpčevská et al. [16] investigated S/L ratios from 1/20 to 1/80 during hydrochloric acid leaching of zinc ash, whereas Huang et al. [83] and Ghasemi and Azizi [84] identified an S/L ratio of approximately 1/20 as the most favorable under their experimental conditions. These differences indicate that the optimum S/L ratio is strongly material- and process-dependent and should therefore be optimized individually according to the mineralogical characteristics of the material, the leaching reagent, and the objectives of the process.

### 3.4. Interphase Contact Time

The effect of interphase contact time on the leaching efficiency of the investigated metals is presented in Figure 6. In general, the leaching efficiency increased with increasing contact time during the initial stage of the process, after which a maximum was reached, indicating that the leaching reactions and processes gradually approached equilibrium.

In the HCl leaching case (Figure 6a), Pb exhibited a non-monotonic leaching profile. The leaching efficiency gradually increased during the initial sampling intervals, reaching a local maximum after 30 min, followed by a temporary decrease. Subsequently, Pb leaching increased again and reached its highest value (approximately 100%) during the final stages of the experiment. This characteristic behavior, with one local extremum, may be attributed to the simultaneous dissolution and transformation of Pb-bearing phases occurring throughout the leaching process. In contrast, Zn and Cu showed a gradual increase in leaching efficiency up to 360 min, after which it remained nearly constant, with only a slight decrease observed at longer contact times. Iron leaching increased steadily during the first 60 min, followed by a stable maximum and a slight increase after 1440 min, suggesting the slower dissolution kinetics of Fe-rich mineral phases.

A similar trend was observed for Pb under the NaOH leaching conditions (Figure 6b), where fluctuations in the process efficiency were also recorded throughout the experiment. In contrast, Fe remained practically insoluble regardless of the interphase contact time. The leaching efficiencies of Zn and Cu increased progressively, reaching their maximum values after approximately 180 min, after which they remained essentially unchanged, indicating that equilibrium was achieved within this period.

For Na_2_NTA solution (Figure 6c), the influence of contact time was less pronounced. The leaching efficiencies of all investigated metals stabilized after approximately 60 min, indicating that extending the contact time beyond this point did not significantly improve metal recovery. This suggests that the complexation reactions between Na_2_NTA and the dissolved metal ions proceeded rapidly and reached equilibrium within a relatively short period; thus, it is not a limiting stage of the process.

Other studies have shown that the optimum leaching conditions strongly depend on both the investigated material and the target metal. For example, optimum extraction times ranging from 20 min for Al and Fe to 60 min for Cu and 90 min for Pb have been reported by Trpčevská et al. [16], whereas Wang et al. [78] identified approximately 120 min as the optimum leaching duration for iron concentrate separated from kiln slag in the leaching process. These findings indicate that no universal leaching conditions can be established, and the process parameters should be optimized individually according to the mineralogical characteristics and chemical composition of the investigated material.

### 3.5. Kinetic Modeling

To identify the kinetic conditions of mass transport phenomena directly influencing the rate of experimentally investigated leaching processes in heterogeneous systems of post-process slag, the experimental data were analyzed using selected theoretical models (2) and (3)–(6).

The application of the Weber–Morris model (2) to analyze the kinetics of metal leaching from slag using the tested leaching agents made it possible to isolate individual stages of the relationship *X* = *f*(*t*^0.5^) and to determine their kinetic parameters *K*_id_ and *C* (Table 1).

Then, to more thoroughly analyze the first stage of the post-process slag leaching, four more detailed kinetic models were independently used, representing theoretically the individual processes or combinations of phenomena controlling the process in its first, fastest stage—under idealized conditions. To identify the potential factors controlling the leaching rate, several theoretical mechanisms were considered and tested (as the experimental data linearization criterion in appropriate coordinates):

- The surface chemical reaction control model (3) (Table 2);
- The diffusion through the product ash layer control model (4) (Table 3);
- The liquid film diffusion control model (5) (Table 4);
- The interface transfer and product diffusion control model (6) (Table 5).

The rates of the subsequent process stages (stages II and III—Table 1) are completely dominated by diffusion transport phenomena in increasingly narrower channels of the porous system—both natural and formed or modified directly during the leaching processes within the slag structure. However, some contribution of specific sequence in time of different chemical species leaching (see sequential analysis Section 3.6.1) should also be considered in the analysis.

Analyzing the effect of HCl as an leaching agent (Table 1), three linear segments of the Weber–Morris model (2) in *X vs. t*^0.5^ coordinates, can be clearly seen. In the first stage of the process (0–30 min), different leaching rates of individual elements can be observed, represented by the different values of the *K*_id_ constant. The Pb is leached the fastest (*K*_id_ = 0.1793 min^−0.5^), whereas Zn the slowest (*K*_id_ = 0.1145 min^−0.5^). Analyzing the values of the *C* constant, which indirectly represents the effect of diffusion resistance in the external liquid boundary layer, the lowest resistance is clearly visible in the case of Cu transport. The next clearly visible linearized segment corresponds to the time window of 60–360 min (Pb, Zn, Cu)—for Fe, it is clearly shifted toward shorter time values of 15–180 min. The *K*_id_ values are significantly reduced by 5–20 times, demonstrating a significant slowdown in the leaching process, primarily due to the systematic narrowing of the transport channels within the solid matrix (mesopore structure). Stage III (micropore structure) can also be identified—under the analyzed process conditions, this corresponds to a time window of 180–1440 min (Zn, Cu, Fe) or 360–1440 min (Pb—a very large reduction in the mass transfer rate). However, the contributing effect of sequential leaching should not be excluded.

When NaOH aqueous solution is used as leaching agent, a wide time span can be observed compared to the first stage of the process. For Pb leaching, this first stage was the shortest (0–30 min), for Zn and Cu it extended up to 0–60 min, and the longest was for Cu leaching (0–180 min). Furthermore, a distinct kinetic selectivity can be observed with respect to the leaching rates of individual metals, expressed by the *K*_id_ values of 0.1846 min^−0.5^ for Pb, 0.0181 min^−0.5^ for Cu, 0.0074 min^−0.5^ for Zn, and 0.0001 min^−0.5^ for Fe. These values slow down significantly in the case of stage II (while maintaining the same relative rate hierarchy). The occurrence of stage III (transport processes in micropores) is clearly noticeable only in the case of Pb leaching—for Zn, Cu and Fe the process is two-stage one (external surface/macropores—mesopores). Effect of sequential leaching can also contribute in this case.

When aqueous solution of Na_2_NTA was used as the leaching agent for all four metals, only two process stages could be distinguished. The first stage, covering a time window of 0–60 min for all studied cases, indicates that the highest leaching rate can be achieved for Pb separation (*K*_id_ = 0.0963 min^−0.5^), while the lowest rate can be expected for Fe leaching (*K*_id_ = 0.0002 min^−0.5^). Intermediate leaching rates were identified for Zn (*K*_id_ = 0.0037 min^−0.5^) and Cu (*K*_id_ = 0.0092 min^−0.5^). Moreover, in the case of Pb and Zn, the second stage of the process (mass transfer in mesopores) starts immediately after the first stage (time window 60–1440 min), while for Fe (180–1440 min) and Cu (360–1440 min) the time windows cover shorter periods of time, which may be related to the extension of the some transition period between stages (no possibility of clear identification of linear segments in the Weber–Morris model (2)).

Analysis of the modeling results, along with SEM, may potentially indicate that the use of aqueous solution of HCl as a leaching agent results in the formation of a more complex porous structure, with three distinctly differentiated time segments (and thus, in terms of pore size—macropores, mesopores, and micropores). This increases the ability of the liquid to penetrate the solid structure and enhances leaching effectiveness—at the cost of significantly extending the necessary interphase contact time. The contribution of the leaching sequence of different metals from different chemicals in the original ore structure should also be considered in analysis due to their different mobilities. The use of other chemicals in the leaching process creates (or modifies) a porous structure with a less differentiated size, which is directly related to the faster process rate.

The first, relatively fast stage of the process, represented in the Weber–Morris model as the first linear segment, identifies the kinetic conditions of the leaching process from the external, easily and directly accessible surface of the solid phase and from the near-surface spatial arrangement of the largest pores of the leached material structure. A non-zero value of the *C* coefficient indicates a certain influence of diffusion resistance in the boundary layer of the liquid phase. Moreover, it clearly suggests that intraparticle diffusion is only one among several potential mechanisms affecting mass transport conditions within the system.

To more precisely identify the phenomena responsible for the complex leaching process, independent but complementary analyses were conducted using four selected kinetic models (3)–(6). Only the first segment of the process was considered, where the direct leaching from the external surface of post-process slag, together with a relatively smaller diffusion resistance contribution in the macropore network, can be roughly comparable to the resistances resulting from other mechanisms determining the intrinsic nature of the leaching process (as indicated by a non-zero *C* value in the Weber–Morris model (2)). An analysis of the kinetic information presented in Table 2, Table 3, Table 4 and Table 5 reveals, however, different leaching mechanisms for the metal–leaching agent combinations.

Using an aqueous HCl solution as the leaching agent, in the case of Pb separation, the dominant and controlling mechanisms are diffusion through the product ash layer (*R*^2^ 0.9983) and surface chemical reaction (*R*^2^ 0.964), while the role of “ash” in the original model is played by the porous structure of the inert residue material, produced (or modified—expanded) during the leaching process. In the case of Cu, model analysis suggests interface transfer and product diffusion control (*R*^2^ 0.9603) as the dominant kinetic mechanism, with the additional influence of liquid film diffusion control (*R*^2^ 0.9066). For the leaching of the other metals, model analysis (Table 2, Table 3, Table 4 and Table 5) does not suggest a clear influence of any of the analyzed potential leaching mechanisms (Zn: *R*^2^ 0.7792–0.9167, Fe: *R*^2^ 0.7599–0.8868).

Changing the leaching agent to an aqueous NaOH solution causes a clear change in the process mechanisms. In the case of Zn, Cu, and Fe separation, relatively high *R*^2^ values suggest the simultaneous occurrence and complementarity of all analyzed kinetic mechanisms of the process. For the surface chemical reaction control model, the *R*^2^ values were 0.9305–0.9859, and for the diffusion through the product ash layer control model, the obtained *R*^2^ values were 0.9128–0.9737. In turn, for the liquid film diffusion control model, the *R*^2^ values for the fit of the experimental data to the model were 0.9261–0.9859, while for the interface transfer and product diffusion control model, the *R*^2^ values were 0.9127–0.9711. On the other hand, relatively low *R*^2^ values (0.7022–0.7283) for Pb did not clearly suggest any of the considered models as the dominant mechanism.

The use of an aqueous Na_2_NTA solution in the leaching process clearly indicates that the Cu and Fe separation processes follow the surface chemical reaction control model (*R*^2^ 0.9949–0.9999) and the liquid film diffusion control model (*R*^2^ 0.9949–1). The complementarity of both mechanisms indicates a more complex leaching process, which cannot be clearly and unambiguously assigned to a single idealized theoretical mechanism. Similarly, in the case of Zn separation, a symmetrical, complementary effect of two theoretical mechanisms can be assumed—it should be noted that these are completely different mechanisms than those statistically identified for Cu and Fe. In this case, the process interaction is well described by both the diffusion through the product ash layer control model (*R*^2^ 0.9786) and the alternative interface transfer and product diffusion control model (*R*^2^ 0.9777). In the case of Pb separation, it was not possible to identify a clearly dominant leaching mechanism due to relatively low fit values observed (*R*^2^ 0.7412–0.9017).

### 3.6. Comparison of Slag Before and After Leaching Process

#### 3.6.1. Sequential Extraction

The sequential three-step BCR extraction results are presented in Figure 7. Figure 7a shows the fractionation of heavy metals in the untreated smelting slag, whereas Figure 7b, Figure 7c and Figure 7d illustrate the distribution of metals after leaching with HCl, NaOH, and Na_2_NTA, respectively.

A comparison of the sequential extraction results demonstrates that the type of leaching agent significantly affected not only the residual concentrations of heavy metals but also their chemical speciation and potential environmental mobility. Distinct redistribution patterns were observed for Pb, Zn, Cu, and Fe depending on the chemistry of the leaching solution. These changes provide information on the potential mobility of the remaining metals; however, they should not be interpreted as a direct assessment of environmental leaching behavior or environmental risk.

Lead exhibited the greatest changes in speciation forms among the investigated metals. In the untreated slag, Pb was predominantly associated with the highly mobile fraction (F1), indicating a relatively high potential mobility under the extraction conditions represented by the BCR procedure. Hydrochloric acid leaching markedly reduced the total Pb concentration. However, the residual lead remained mainly associated with chemical species within the F1 fraction, suggesting that the remaining Pb was still present in readily mobile forms that could be easily released into the environment, highlighting the need for further work to improve the leaching efficiency. In contrast, alkaline leaching with NaOH solution promoted the stabilization (immobilization) of Pb by increasing its proportion in the residual fraction (F4), suggesting a shift toward less readily extractable forms according to the BCR classification. Increasing the degree of leaching of the most mobile fractions during the process systematically changes the original proportions between fractions of different mobility. Following Na_2_NTA treatment, Pb was distributed mainly between the potentially mobile (F2) and residual (F4) fractions, with a substantial contribution from the exchangeable fraction (F1). This distribution differed from that observed after HCl and NaOH treatment, indicating that Na_2_NTA affected the post-leaching fractionation of Pb differently from the acidic and alkaline leaching systems.

The speciation of zinc also depended strongly on the applied leaching reagent. In the untreated slag, Zn was predominantly associated with the residual fraction, reflecting its occurrence in relatively stable mineral phases. After HCl leaching, the remaining Zn was mainly redistributed into the potentially mobile fraction (F2), suggesting preferential dissolution of more stable Zn-bearing phases and retention of Zn associated with reducible components. In contrast, both NaOH and Na_2_NTA solutions maintained zinc predominantly in the residual fraction, indicating that most of the remaining Zn occurred in forms classified as less readily extractable by the BCR procedure.

Following leaching, copper remained predominantly associated with the less mobile fractions (F3 and F4), whereas the combined contribution of the mobile fractions (F1 and F2) remained negligible under all investigated conditions. In the untreated slag, Cu occurred mainly in the residual fraction (F4), indicating its incorporation into highly stable mineral phases. Following hydrochloric acid leaching, the remaining copper was found predominantly in the potentially immobile fraction (F3). In contrast, both NaOH and Na_2_NTA solutions resulted in more than 90% of the residual Cu remaining associated with the combined F3 and F4 fractions, indicating a predominance of less readily extractable Cu forms according to the BCR classification.

Iron showed the smallest changes in speciation regardless of the leaching agent. In all treated samples, Fe was predominantly retained in fraction F4, indicating that most of the remaining Fe was associated with forms classified as residual by the BCR procedure. Only minor proportions of Fe were detected in the mobile fractions, indicating that iron-containing phases were largely resistant to chemical dissolution.

The sum of the four BCR fractions accounted for 93% of the total Pb and Zn contents, 103% of the Cu content, and 89% of the Fe content determined independently by digestion for the untreated slag. These recoveries indicate generally good agreement between the sequential extraction and total metal determination, although the lower recovery observed for Fe may reflect incomplete extraction and/or analytical variability associated with the sequential procedure.

Overall, the BCR results indicate that the effect of the leaching agent extended beyond the reduction of total metal concentrations and was also reflected in changes in the operationally defined distribution of the residual metals. Hydrochloric acid resulted in substantial Pb removal but left a considerable proportion of the remaining Pb in the F1 fraction, whereas NaOH resulted in a greater proportion of residual Pb being associated with fraction F4. Na_2_NTA produced an intermediate distribution, with Pb occurring mainly in fractions F2 and F4. For Zn, Cu, and Fe, the majority of the residual metals remained associated with the less readily extractable fractions under the most investigated conditions. These findings indicate differences in the potential mobility of residual metals following treatment; however, BCR fractionation alone does not constitute a standardized environmental leaching or risk assessment. Confirmation of the environmental behavior of the treated residues would require dedicated standardized leaching tests and/or further environmental assessment.

Comparison with previous studies demonstrates that the distribution of metals among BCR fractions strongly depends on the investigated material. Sungur et al. [25] reported that Cu and Zn in contaminated soils were predominantly associated with the residual fraction, whereas Pb was distributed among all fractions, with a considerably lower contribution of the exchangeable fraction than observed in the present study. In contrast, Liu et al. [29] found that Pb, Zn, and Fe in copper smelting slags occurred predominantly in the residual fraction, while Cu exhibited a substantially higher contribution of the exchangeable and reducible fractions. These differences indicate that metal speciation is controlled primarily by the mineralogical composition and mode of metal occurrence within the investigated waste rather than by the metal itself. Consequently, the leaching behavior and environmental mobility of heavy metals cannot be predicted solely on the basis of their total concentrations; their geochemical distribution among individual fractions should also be considered.

#### 3.6.2. Scanning Electron Microscopy (SEM) Analysis

The morphological characteristics of the tested slag before and after leaching with 2 mol/dm^3^ HCl solution are presented in Figure 8. The micrographs were obtained using scanning electron microscopy (SEM) at magnifications of 500× and 1000×.

The untreated slag (Figure 8a,c) predominantly consists of dense, angular particles with irregular shapes and relatively smooth, compact surfaces. The particles exhibit a broad size distribution, with coarse fragments surrounded by a fine-grained matrix. The overall morphology indicates a mechanically fragmented material with limited surface deterioration and without noticeable porosity. No significant dissolution features are observed on the particle surfaces prior to the leaching process.

In contrast, the leached sample (Figure 8b,d) exhibits significant morphological changes resulting from HCl leaching. Numerous particles are characterized by a highly porous structure with irregular shapes and interconnected surfaces, indicating the intense impact of HCl. The surfaces of larger particles become noticeably rougher and show evidence of localized dissolution. In addition, several particles appear partially fragmented, suggesting that the leaching process weakened the original structural integrity of the slag matrix.

The observed morphological evolution is consistent with the selective dissolution of acid-soluble mineral phases. Their removal during the leaching process promoted the development of secondary porosity and extensive surface deterioration. The higher magnification images (Figure 8c,d) further emphasize the extent of surface modification. Whereas the untreated particles retained relatively smooth and compact surfaces, the leached particles exhibited pronounced surface roughening, pore formation, and localized structural degradation. These observations provide direct evidence of the effectiveness of HCl in modifying the slag microstructure through the dissolution of reactive mineral phases. The observed changes are further supported by the BCR sequential extraction results, which revealed a substantial depletion of the most mobile metal fractions following HCl leaching. Moreover, the formation of a porous residual structure is in agreement with the kinetic analysis, which indicated that Pb leaching was controlled by a combination of a surface chemical reaction and diffusion through the product layer, suggesting that progressive dissolution of reactive phases generated an increasingly porous diffusion barrier during the leaching process.

## 4. Materials and Methods

### 4.1. Sample Collection and Processing

The material subjected to leaching tests was slag from the Short Rotary Kiln of the Lead Refining Department at the Zinc Smelter in Poland. The material was collected directly from the Department’s production line. Before subjecting the sample to heavy metal leaching, some mechanical pre-treatment of the material was done (crushing and milling). Subsequently, the sample was dried up to constant mass stabilization, ground in a ball mill (S1000, Retsch, Verder Polska Sp. z o.o., Wroclaw, Poland), and then sieved through a 0.06 mm mesh. The total metal content (Ca, Cd, Cu, Fe, K, Mg, Mn, Na, Pb, and Zn) was determined after microwave-assisted *aqua regia* digestion (MLS 1200 Mega High Digestion Unit, Milestone, Spectro-Lab, Warsaw, Poland). The mixture was analyzed by flame atomic absorption spectroscopy (AAS) (SpectrAA-880, Varian, Candela Sp. z o.o., Warsaw, Poland). Standard solutions of each metal (1000 mg/dm^3^, Merck Life Science Sp. z o.o., Poznan, Poland) were used for spectrophotometer calibration.

In addition, an X-ray diffraction (XRD) analysis was conducted to qualitatively identify the crystalline phases present in the tested material (diffractometer X’PERT PRO, PANanalytical, Warsaw, Poland). Due to the presence of a significant amorphous phase, quantitative phase analysis was not performed.

### 4.2. Solid–Liquid Leaching for Heavy Metals Recovery

The leaching tests involved the analysis of a number of process parameters:

- type and concentration of the leaching agent (HCl, NaOH, Na_2_NTA);
- pH (for Na_2_NTA);
- solid-to-liquid ratio (S/L);
- interphase contact time;

and their impact on the leaching efficiency of four metals: Pb, Zn, Cu, and Fe.

A single leaching test involved placing 0.5 g of a dried, ground, and sieved slag sample into a glass container and adding 50 cm^3^ of leaching agent at a specific concentration, which ensured a solid-to-liquid ratio (S/L) of 1/100. The suspension was then shaken for 24 h (Orbital Shaker SK-0330-Pro, Chemland, Stargard, Poland), filtered through a 0.45 µm cellulose acetate syringe membrane filter (CA 25 mm 0.45 µm, Chemland, Stargard, Poland), and analyzed for identification of Pb, Zn, Cu, and Fe content. Each experimental condition was tested in triplicate using three individually prepared slag samples and independently conducted leaching procedures. The results are presented as mean values, with error bars representing the standard deviation (SD) of the three independent experiments.

Process efficiency (*X*) was calculated as

(6) $$X=\frac{{m\;}_{leached}}{{m\;}_{in\;slag}}\cdot 100\%$$

and determined for each leaching agent–metal combination based on the ratio of the mass of metal leached from one gram of slag (*m_leached_*) to its total content in one gram of material (*m_in slag_*), as determined after digestion.

#### 4.2.1. Effect of Leaching Agent Type and Concentration

This study investigated the effect of the type and concentration of the leaching agent on the leaching efficiency of Pb, Zn, Cu, and Fe from the slag sample under study. For the tests, one leaching agent was selected from each of the following groups: acids, bases, and complexing agents. These were, respectively, HCl (35–38%, Stanlab, Lublin, Poland), NaOH (97%, Merck Life Science Sp. z o.o., Poznan, Poland), and Na_2_NTA (≥98%, Merck Life Science Sp. z o.o., Poznan, Poland).

The following concentrations were used during the tests:

- HCl—0.05, 0.1, 0.25, 0.5, 1.0, 1.5, 2.0, 2.5 mol/dm^3^;
- NaOH—0.25, 0.5, 1.0, 1.5, 2.0 mol/dm^3^;
- Na_2_NTA—0.001, 0.005, 0.01, 0.02, 0.04, 0.08 mol/dm^3^.

The other process parameters used during the test were uncorrected pH, S/L = 1/100, and time = 24 h.

#### 4.2.2. Effect of Complexing Agent pH

The pH of the environment in which the leaching process takes place is significant in the context of using complexing leaching agents. This parameter affects the stability of the mobile metal–complex compound formed during the process and also affects the solubility of the compounds. In this study, a 0.005 mol/dm^3^ Na_2_NTA leaching solution was used, and the pH was varied as follows: 2, 5, 8, 10, and 12. The pH adjustment was performed using HNO_3_ (J.T.Baker, Avantor, Gliwice, Poland) or NaOH (Merck Life Science Sp. z o.o., Poznan, Poland) aqueous solutions. The other process parameters used during the test were S/L = 1/100 and time = 24 h.

#### 4.2.3. Effect of Solid-to-Liquid (S/L) Ratio

The solid-to-liquid (S/L) ratio was another process parameter evaluated in terms of its effect on metal leaching efficiency. The S/L ratio was defined as the ratio of the mass of the solid slag sample to the volume of the leaching solution. This parameter affects the relative amount of metal-bearing material in relation to the available amount of leaching agent and may therefore influence the efficiency of the leaching process. In this study, S/L ratios of 1/50, 1/100, and 1/200 were investigated. For each ratio, the initial mass of the slag sample was adjusted while maintaining a constant volume of the leaching solution. Thus, for the S/L ratio of 1/100, 0.5 g of slag was leached with 50 mL of leaching solution. The other process parameters were kept constant: concentration, 2 mol/dm^3^ HCl; 0.005 mol/dm^3^, pH = 8 Na_2_NTA; 2 mol/dm^3^ NaOH; and time = 24 h.

#### 4.2.4. Effect of Interphase Contact Time

The study of the effect of interphase contact time on metal leaching efficiency was a key factor, the basis for further analyzing the kinetics of the leaching process. The process was carried out for the following durations: 15, 30, 60, 180, 360, 960, and 1440 min. The other parameters used during the test were concentration, 2 mol/dm^3^ HCl; 0.005 mol/dm^3^, pH = 8 Na_2_NTA; 2 mol/dm^3^ NaOH; and S/L = 1/100.

### 4.3. Sequential Extraction

The slag sample (1.0000 ± 0.0034 g, dry mass) was subjected to the modified three-step sequential extraction procedure proposed by the *Community Bureau of Reference* (BCR). Quevauviller et al. [85] originally developed the BCR sequential extraction scheme to harmonize the fractionation of trace metals in soils and sediments, while Rauret et al. [28] subsequently improved and standardized the procedure. Such a modified protocol was applied in this study, followed by digestion of the residual fraction.

Initially, 40 cm^3^ of 0.11 mol/dm^3^ acetic acid (glacial, Merck Life Science Sp. z o. o., Poland) was added to the sample to extract the acid-soluble and exchangeable fraction (F1). The suspension was shaken for 16 h at room temperature and subsequently centrifuged (centrifuge MPW-380, MPW MED. INSTRUMENTS, Warsaw, Poland) for 20 min at 3000 rpm to separate the liquid and solid phases. The solid residue was washed with deionized water, shaken for 15 min, centrifuged (3000 rpm, 20 min), and separated from the washing solution before proceeding to the next stage.

Then, the reducible fraction (F2) was extracted using 40 cm^3^ of 0.5 mol/dm^3^ hydroxylamine hydrochloride (Warchem, Zakret, Poland) adjusted to pH 1.5 with nitric acid (65%, J. T. Baker, Avantor, Gliwice, Poland). After extraction for 16 h at room temperature, the suspension was centrifuged (3000 rpm, 20 min), the supernatant was collected, and the solid residue was washed with deionized water (as previously described).

For the oxidizable fraction (F3), the residue was treated with 10 cm^3^ of hydrogen peroxide (8.8 mol/dm^3^, Chempur, Piekary Slaskie, Poland) and allowed to react for 1 h at room temperature. The suspension was then heated to 80 °C for 1 h with occasional manual agitation until the volume was reduced to a few milliliters. After cooling, another portion of 10 cm^3^ hydrogen peroxide was added, and the heating procedure was repeated. After oxidation and evaporation to near dryness, 50 cm^3^ of 1 mol/dm^3^ ammonium acetate (Chempur, Piekary Slaskie, Poland) adjusted to pH 2 with nitric acid was added to extract metals associated with oxidizable phases. Following extraction, the suspension was centrifuged, and the residue was washed with deionized water, as described above.

The remaining solid residue was digested by microwave-assisted *aqua regia* digestion (HCl:HNO_3_, 3:1 (*v*/*v*)) to determine the residual fraction (F4).

Following each extraction stage, the collected leachates were acidified with 2 cm^3^ of concentrated nitric acid and analyzed for Pb, Zn, Cu, and Fe concentrations using atomic absorption spectrometry (AAS). The total metal content in the slag sample was calculated as the sum of the concentrations determined in fractions F1–F4. The completeness of the sequential extraction procedure was assessed for the untreated slag by comparing the sum of metals recovered in fractions F1-F4 with the independently determined total metal concentration obtained after digestion. For comparative purposes, the initial slag samples, as well as the solid residues obtained after leaching with each leaching agents conducted at chosen process parameters (2 mol/dm^3^ HCl; 0.005 mol/dm^3^, pH = 8 Na_2_NTA; 2 mol/dm^3^ NaOH, S/L = 1/100, contact time = 24 h) were characterized.

### 4.4. SEM Analysis

In order to characterize the morphology and externally visible microstructure of the slag, and to determine the specific chemical composition of selected microareas, the sample was subjected to SEM imaging using a scanning electron microscope coupled with energy-dispersive X-ray spectroscopy (SEM-EDS) (Supra 35, Carl Zeiss, Jena, Germany). The analyses were performed on representative slag particles without prior grinding in a ball mill or sieving in order to preserve their original morphology and phase distribution. Due to the high heterogeneity of the slag samples, the analysis was conducted at several locations on different grains, and selected, representative images are discussed. For comparison, identical analyses were performed on slag sample after leaching with a 2 mol/dm^3^ HCl aqueous solution to assess changes in morphology and phase composition resulting from the leaching process.

## 5. Conclusions

Based on the experimental results and kinetic analysis, the following main conclusions can be drawn regarding the leaching behavior of Pb, Zn, Cu, and Fe from the investigated zinc–lead metallurgical slag:

1. The investigated zinc–lead metallurgical slag was characterized by a heterogeneous mineralogical and chemical composition, with Pb, Zn, Cu, and Fe occurring in phases differing in their stability and susceptibility to dissolution. The results demonstrate that the leaching behavior of these metals is strongly material-specific and cannot be predicted from their total concentrations alone.
2. The comparative evaluation of acidic (HCl), alkaline (NaOH), and complexing (Na_2_NTA) leaching systems demonstrated substantial differences in both metal leaching efficiency and selectivity. Hydrochloric acid provided the highest simultaneous extraction of Pb, Zn, and Cu, exceeding 90% under the selected conditions, whereas NaOH and Na_2_NTA showed greater selectivity, particularly toward Pb. Thus, the choice of leaching system should be considered together with the target metal and the desired degree of selectivity rather than based solely on the overall extraction efficiency.
3. The investigated process parameters—leaching agent concentration, solid-to-liquid ratio, contact time, and, for Na_2_NTA, solution pH—affected metal leaching to different extents depending on the metal–leaching agent combination; therefore, the optimization of the leaching process should not be generalized for different metals, even for the same leaching agent, due to their potentially different behaviors (e.g., the different responses of Pb and Zn to pH changes when using Na_2_NTA).
4. The modified BCR sequential extraction demonstrated that the observed leaching behavior was closely related to the operationally defined chemical distribution of the metals. Pb was predominantly associated with the more mobile fractions, whereas Cu and Fe were mainly present in the residual fraction. Comparison of untreated and leached residues further showed changes in the relative distribution of metals among the BCR fractions, supporting the use of sequential extraction as a complementary tool for interpreting leaching behavior and assessing changes in potential metal mobility.
5. The kinetic analysis demonstrated that no single kinetic model generally described the leaching behavior of all investigated metal–leaching agent combinations. Based on the results of the kinetic modeling, it can be inferred that no single kinetic mechanism was consistently dominant across the investigated systems. This may be related to the diverse physicochemical interactions between the leaching agents and the individual metals present in the complex slag matrix. The contribution of intraparticle diffusion was particularly evident in the HCl system, while the relative contribution of the kinetic stages varied with the metal–leaching agent. The time-dependent experiments further indicated that the most pronounced increase in leaching efficiency generally occurred during the first several hours, with the leaching curves approaching a plateau at approximately 400 min in most investigated systems. This indicates the potential for shortening the contact time compared with the 24 h experimental period, although the most favorable time should be established individually for each metal–leaching agent combination.
6. The integrated evaluation of mineralogical characteristics, leaching efficiency, process parameters, kinetic behavior, and post-leaching metal speciation demonstrated that the response of Pb, Zn, Cu, and Fe to a given leaching system cannot be explained solely by their total concentrations in the slag. The comparative results showed that both the effectiveness and the apparent controlling mechanisms of leaching depended on the specific metal and the chemistry of the leaching system. The systematic comparison of acidic, alkaline, and complexing leaching systems under different process conditions, combined with metal-specific kinetic analysis and post-leaching speciation, provides a comprehensive basis for understanding the complex and metal-specific leaching behavior of zinc–lead metallurgical slags.

## Figures and Tables

**Figure 1:**
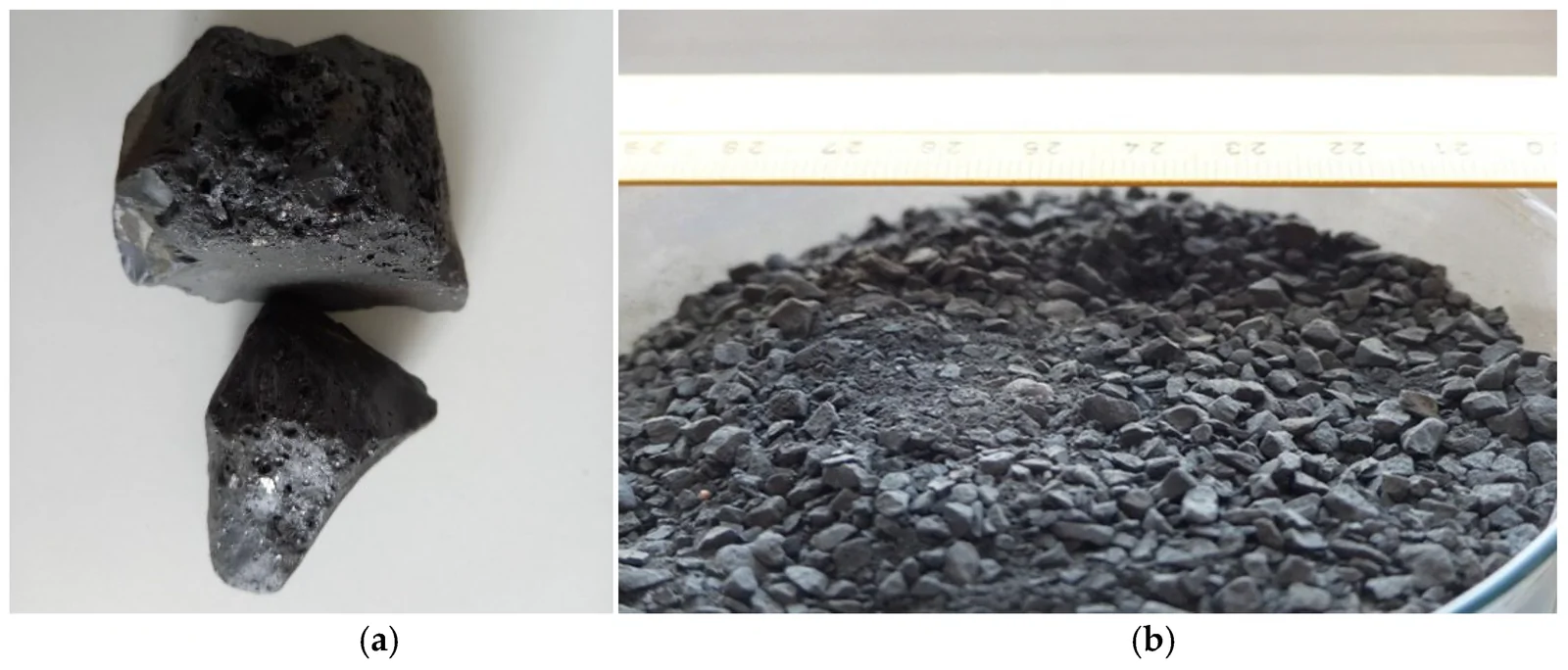
(**a**) Slag collected from the smelter; (**b**) slag after preliminary processing.

**Figure 2:**
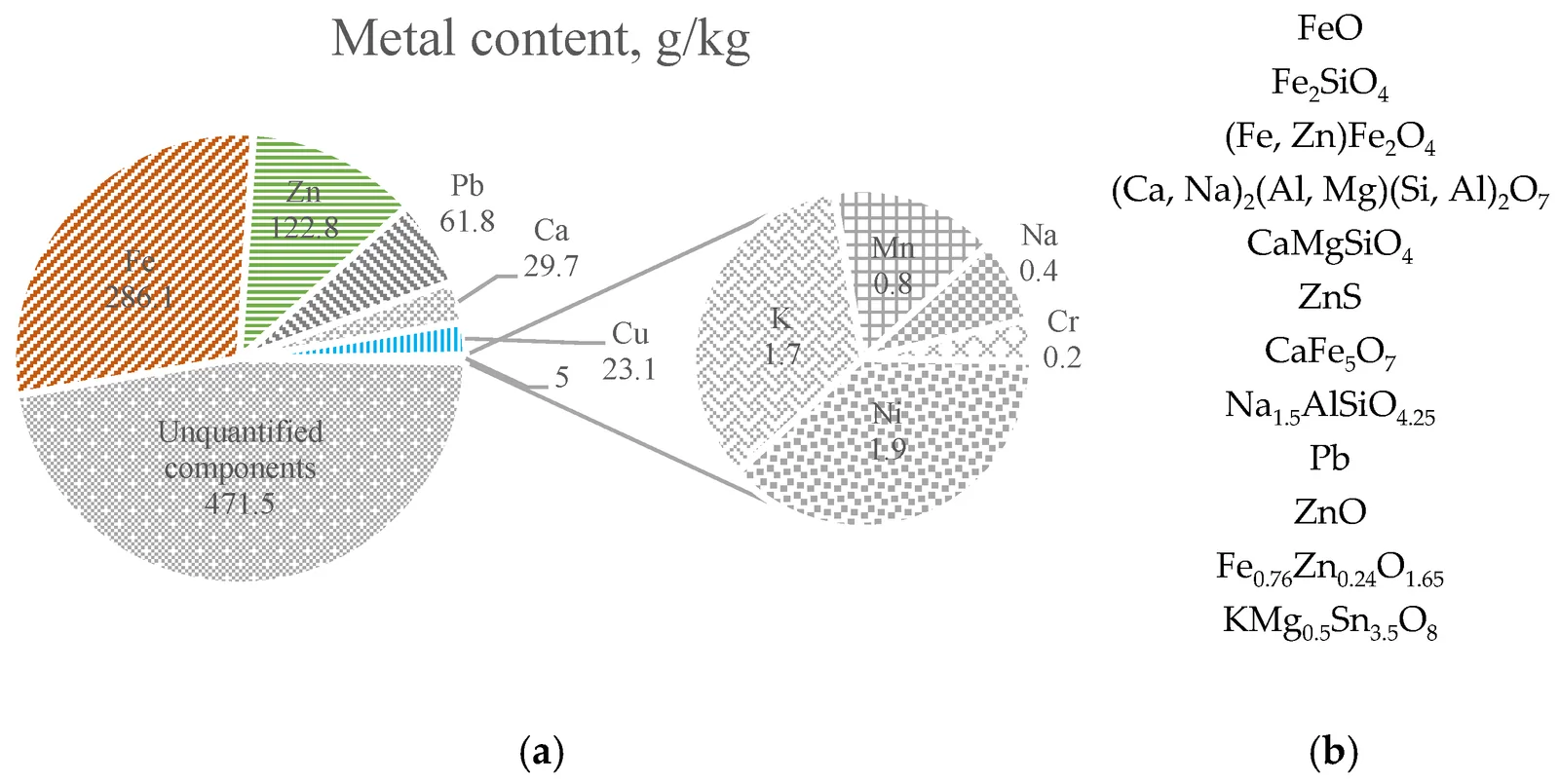
(**a**) Metal concentrations in the investigated slag determined by AAS. (**b**) Crystalline phase identified by XRD analysis in the tested material.

**Figure 3:**
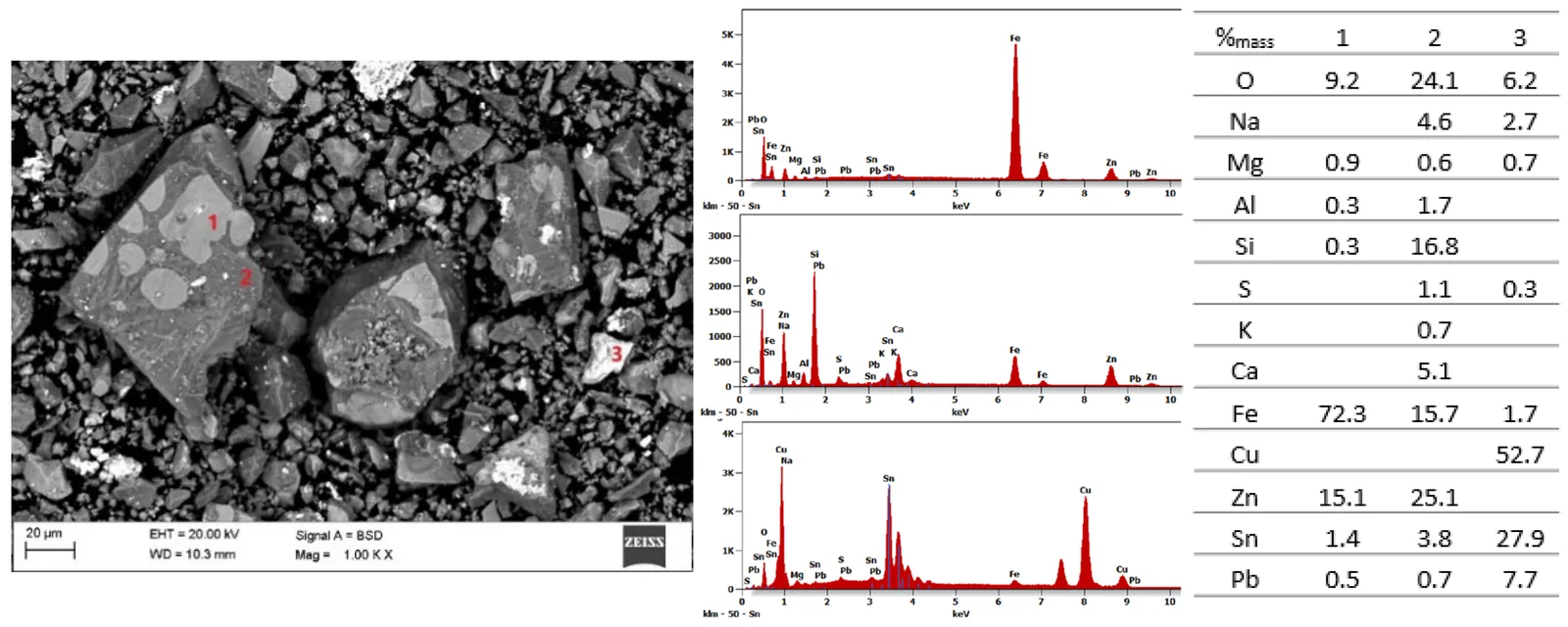
Exemplary SEM analysis (1000× magnification) with EDS spectra and measurements for selected sample points (1–3).

**Figure 4:**
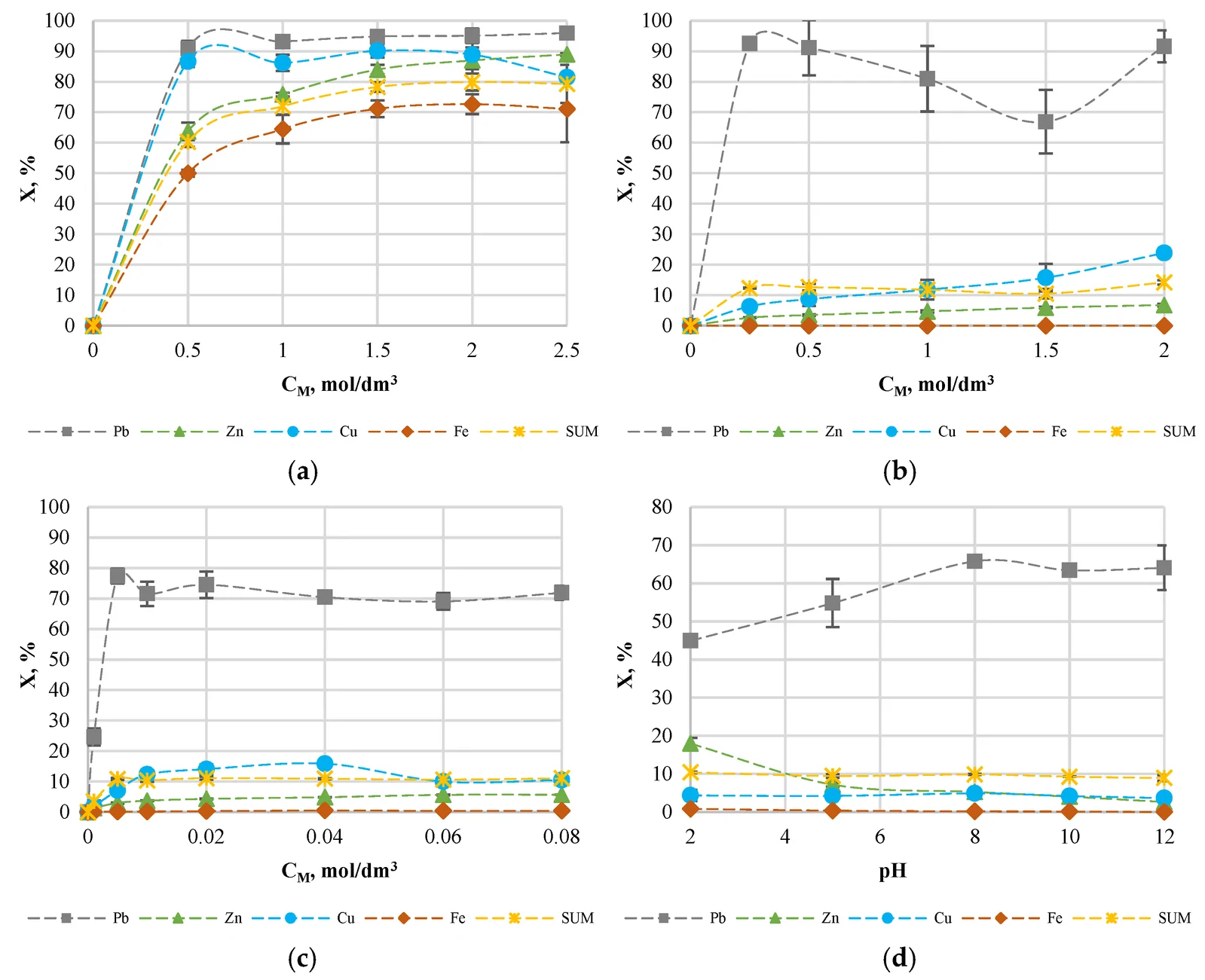
Metal leaching efficiency (X) as a function of leaching agent concentration (C_M_): (**a**) HCl, (**b**) NaOH, (**c**) Na_2_NTA aqueous solutions, and (**d**) pH of the Na_2_NTA solution.

**Figure 5:**
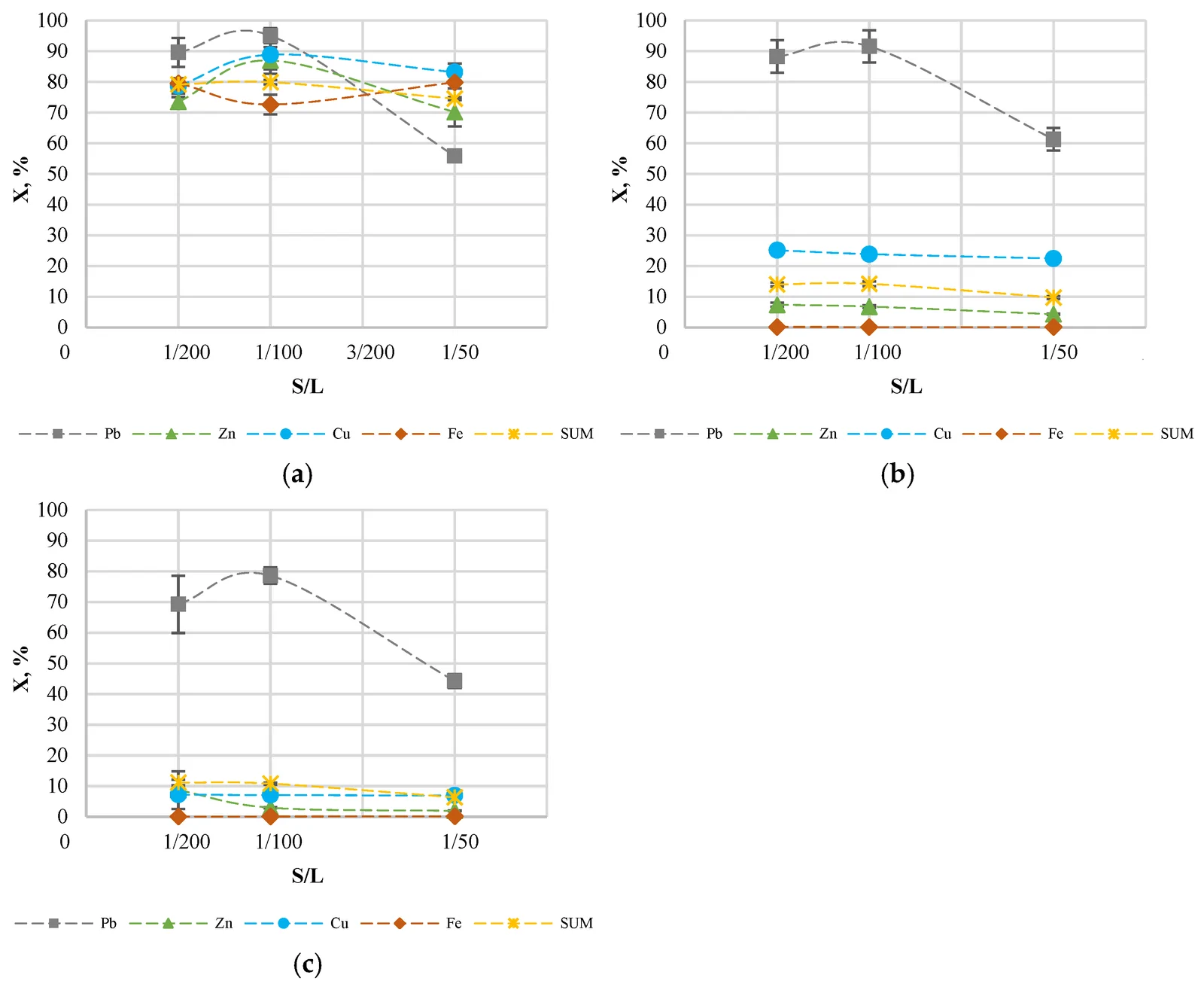
Metal leaching efficiency (X) as a function of solid-to-liquid (S/L) ratio: (**a**) HCl, (**b**) NaOH, and (**c**) Na_2_NTA aqueous solutions.

**Figure 6:**
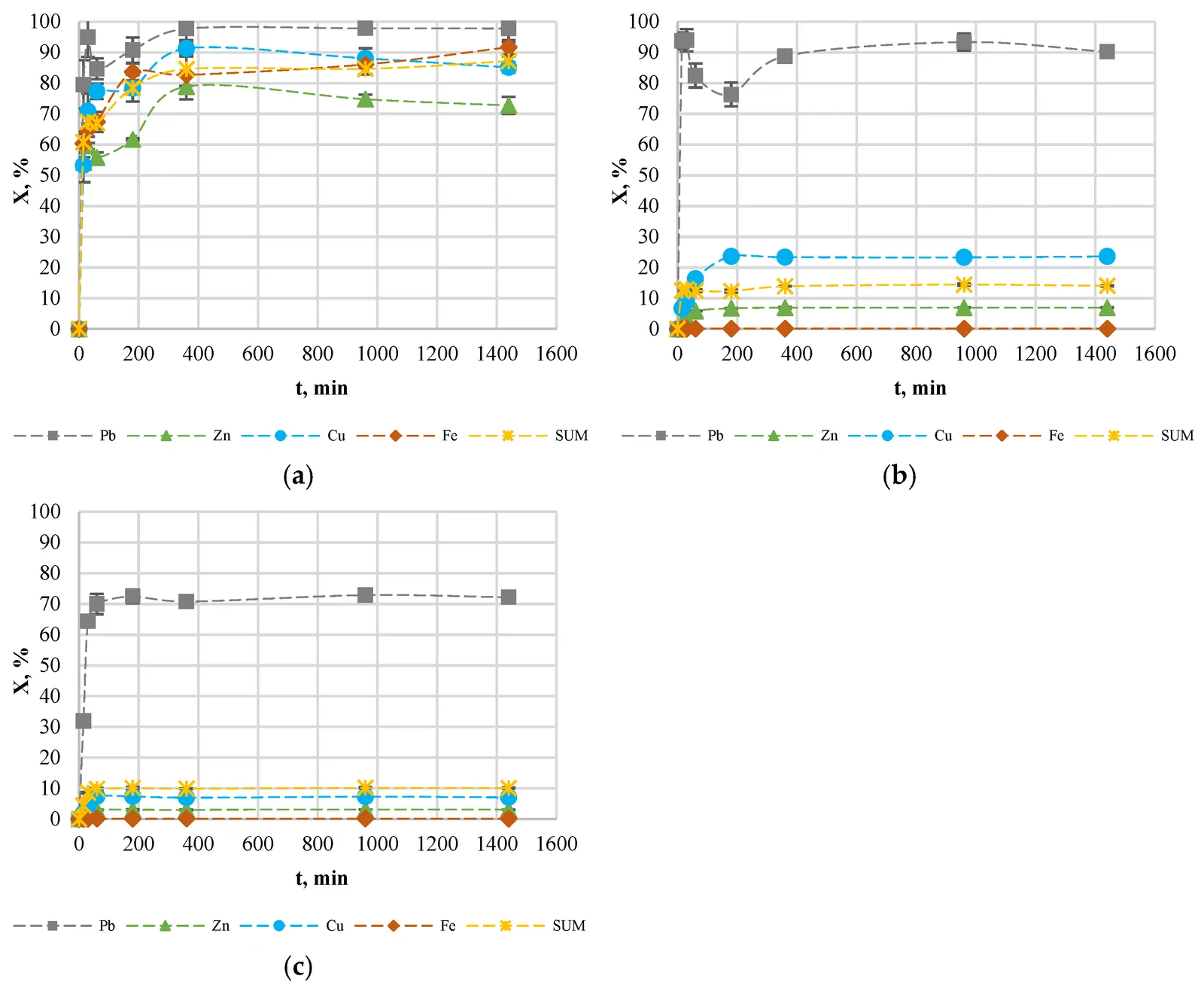
Metal leaching efficiency (X) as a function of contact time (t): (**a**) HCl, (**b**) NaOH, and (**c**) Na_2_NTA aqueous solutions.

**Figure 7:**
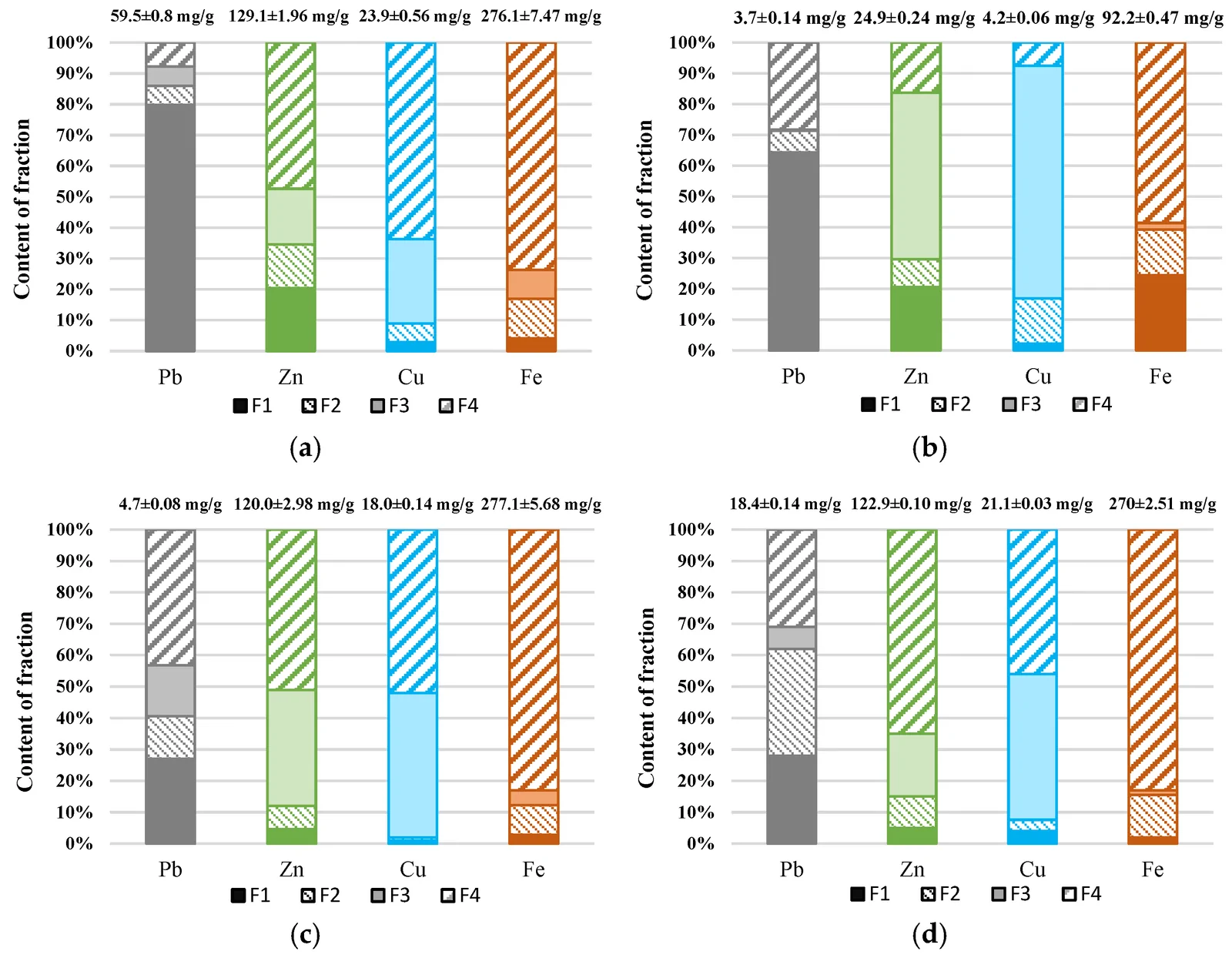
Distribution of Pb, Zn, Cu, and Fe in the defined fractions (F1–F4) determined by the BCR sequential extraction procedure for the untreated slag sample (**a**) and the slag samples after leaching with HCl (**b**), NaOH (**c**), and Na_2_NTA (**d**) aqueous solutions. Values shown above the bars indicate the total metal content determined for the respective sample, corresponding to 100% of the analyzed metal.

**Figure 8:**
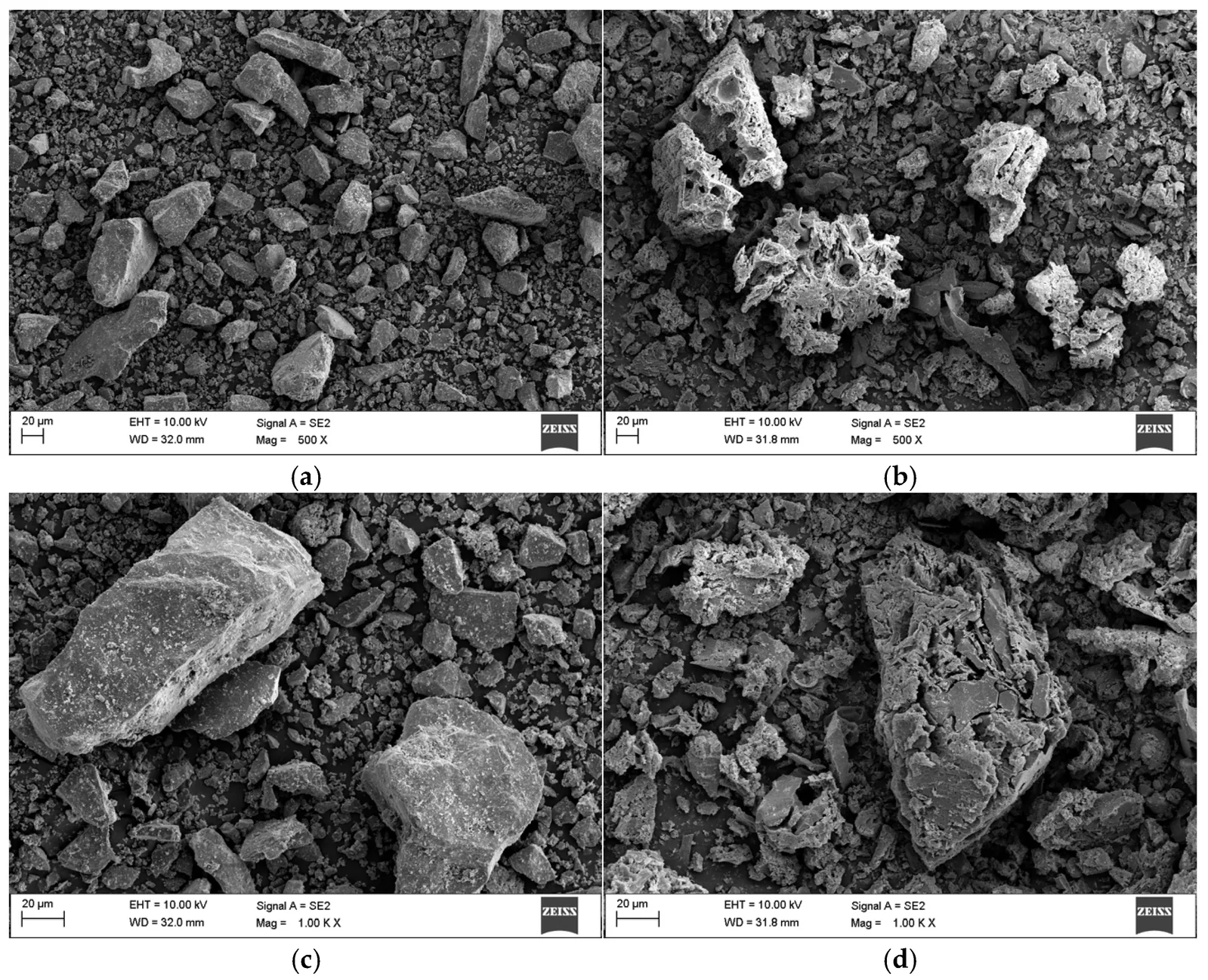
SEM images of the sample before leaching: (**a**) 500× zoom, (**c**) 1000× zoom; sample after leaching: (**b**) 500× zoom, (**d**) 1000× zoom.

**Table 1:** The kinetic calculation results—application of the Weber–Morris model to experimental data indicating kinetic regions where different mass transfer mechanisms dominate (segments).

	Weber–Morris Model									
	Leached Metal	Leaching Agent								
		Stage I			Stage II			Stage III		
		*K*_id_, min^−0.5^	*C*	R^2^	*K*_id_, min^−0.5^	*C*	R^2^	*K*_id_, min^−0.5^	*C*	R^2^
		HCl								
		0–30 min			60–360 min			360–1440 min		
1	Pb	0.1793	0.023	0.9812	0.0116	0.7547	0.998	0.00002	0.9771	0.1658
		0–30 min			60–360 min			180–1440 min		
2	Zn	0.1145	0.0224	0.9571	0.0206	0.3799	0.9231	0.0028	0.6501	0.1773
		0–30 min			60–360 min			180–1440 min		
3	Cu	0.1309	0.0058	0.9977	0.0124	0.6578	0.8004	0.0015	0.8182	0.0979
		0–30 min			15–180 min			180–1440 min		
4	Fe	0.1251	0.027	0.9485	0.0242	0.5062	0.984	0.0032	0.7781	0.8052
		NaOH								
		0–30 min			60–360 min			360–1440 min		
5	Pb	0.1846	0.0504	0.9200	0.0055	0.7513	0.2478	0.0011	0.8757	0.201
		0–60 min			180–1440 min			min		
6	Zn	0.0074	−0.0017	0.9878	0.00006	0.0673	0.5230	-	-	-
		0–180 min			360–1440 min			min		
7	Cu	0.0181	0.001	0.9802	0.0001	0.2311	0.4415	-	-	-
		0–60 min			180–1440 min			min		
8	Fe	0.0001	−0.0001	0.8993	0.0000002	0.0013	0.1713	-	-	-
		Na_2_NTA								
		0–60 min			60–1440 min			min		
9	Pb	0.0963	0.0038	0.9414	0.0006	0.7027	0.4100	-	-	-
		0–60 min			60–1440 min			min		
10	Zn	0.0037	−0.0002	0.9889	0.00003	0.0297	0.6528	-	-	-
		0–60 min			360–1440 min			min		
11	Cu	0.0092	−0.007	0.9035	0.00008	0.0687	0.2017	-	-	-
		0–60 min			180–1440 min			min		
12	Fe	0.0002	−0.0001	0.913	0.000003	0.0011	0.1456	-	-	-

**Table 2:** The kinetic calculation results—application of the surface chemical reaction control model to experimental data.

	The Surface Chemical Reaction Control Model						
	Leached Metal	Leaching Agent					
		HCl		NaOH		Na_2_NTA	
		*k*_1_, min^−1^	R^2^	*k*_1_, min^−1^	R^2^	*k*_1_, min^−1^	R^2^
		0–30 min		0–30 min		0–60 min	
1	Pb	0.0223 (0.021)	0.964 (0.970)	0.0243 (0.0203)	0.7074 (0.7561)	0.0064 (0.0055)	0.813 (0.851)
		0–30 min		0–60 min		0–60 min	
2	Zn	0.010 (0.0087)	0.8051 (0.8376)	0.0004 (0.0003)	0.9305 (0.9583)	0.0002 (0.0002)	0.8731 (0.9226)
		0–60 min		0–60 min		0–60 min	
3	Cu	0.0078 (0.0061)	0.6876 (0.8031)	0.001 (0.0009)	0.9589 (0.9737)	0.0004 (0.0004)	0.9999 (0.9999)
		0–30 min		0–60 min		0–60 min	
4	Fe	0.0114 (0.0098)	0.7844 (0.8203)	0.000006 (0.000006)	0.9859 (0.9860)	0.000007 (0.000007)	0.9949 (0.9950)

Note: Data presentation format: kinetic constant *k*_1_ calculated based on the theory-based linear correlation form y = a × x (additional information for comparison is given in brackets—kinetic constant *k*_1_ calculated based on alternative correlation form y = a × x + b with a higher R^2^).

**Table 3:** The kinetic calculation results—application of the diffusion through the product ash layer control model to experimental data.

	The Diffusion Through the Product Ash Layer Control Model						
	Leached Metal	Leaching Agent					
		HCl		NaOH		Na_2_NTA	
		*k*_2_, min^−1^	R^2^	*k*_2_, min^−1^	R^2^	*k*_2_, min^−1^	R^2^
		0–30 min		0–30 min		0–60 min	
1	Pb	0.0233 (0.023)	0.9983 (0.9986)	0.0263 (0.022)	0.7097 (0.7581)	0.0047 (0.0045)	0.8767 (0.8779)
		0–30 min		0–60 min		0–60 min	
2	Zn	0.0063 (0.0056)	0.8743 (0.8953)	0.00002 (0.00002)	0.9737 (0.9815)	0.000005 (0.000005)	0.9786 (0.9804)
		0–60 min		0–60 min		0–60 min	
3	Cu	0.0064 (0.0056)	0.876 (0.9089)	0.0001 (0.0002)	0.9361 (0.9525)	0.00003 (0.00003)	0.8795 (0.9214)
		0–30 min		0–60 min		0–60 min	
4	Fe	0.0079 (0.0069)	0.841 (0.8675)	0.000000005 (0.000000006)	0.9128 (0.9475)	0.000000007 (0.000000008)	0.9082 (0.9434)

Note: Data presentation format: kinetic constant *k*_2_ calculated based on the theory-based linear correlation form y = a × x (additional information for comparison is given in brackets—kinetic constant *k*_2_ calculated based on alternative correlation form y = a × x + b with a higher R^2^).

**Table 4:** The kinetic calculation results—application of the liquid film diffusion control model to experimental data.

	The Liquid Film Diffusion Control Model						
	Leached Metal	Leaching Agent					
		HCl		NaOH		Na_2_NTA	
		*k*_3_, min^−1^	R^2^	*k*_3_, min^−1^	R^2^	*k*_3_, min^−1^	R^2^
		0–30 min		0–30 min		0–60 min	
1	Pb	0.0359 (0.0316)	0.8413 (0.8678)	0.0376 (0.0313)	0.7022 (0.7518)	0.014 (0.0114)	0.7412 (0.8128)
		0–30 min		0–60 min		0–60 min	
2	Zn	0.0231 (0.0198)	0.7792 (0.816)	0.0011 (0.0009)	0.9261 (0.9557)	0.0005 (0.0005)	0.8702 (0.9209)
		0–30 min		0–60 min		0–60 min	
3	Cu	0.026 (0.0236)	0.9066 (0.9221)	0.0029 (0.0026)	0.9495 (0.9683)	0.0012 (0.0012)	1 (1)
		0–30 min		0–60 min		0–60 min	
4	Fe	0.0253 (0.0216)	0.7599 (0.7999)	0.00002 (0.00002)	0.9859 (0.9859)	0.00002 (0.00002)	0.9949 (0.995)

Note: Data presentation format: kinetic constant *k*_3_ calculated based on the theory-based linear correlation form y = a × x (additional information for comparison is given in brackets—kinetic constant *k*_3_ calculated based on alternative correlation form y = a × x + b with a higher R^2^).

**Table 5:** The kinetic calculation results—application of the interface transfer and product diffusion control model to experimental data.

	The Interface Transfer and Product Diffusion Control Model						
	Leached Metal	Leaching Agent					
		HCl		NaOH		Na_2_NTA	
		*k*_4_, min^−1^	R^2^	*k*_4_, min^−1^	R^2^	*k*_4_, min^−1^	R^2^
		0–30 min		0–30 min		0–60 min	
1	Pb	0.0212 (0.0236)	0.899 (0.9159)	0.0244 (0.0205)	0.7283 (0.7736)	0.0016 (0.0017)	0.9017 (0.9026)
		0–30 min		0–60 min		0–60 min	
2	Zn	0.0018 (0.0017)	0.9167 (0.9306)	0.000003 (0.000003)	0.9711 (0.9797)	0.0000008 (0.0000008)	0.9777 (0.9797)
		0–60 min		0–60 min		0–60 min	
3	Cu	0.0026 (0.0025)	0.9603 (0.9629)	0.00003 (0.00003)	0.9255 (0.9451)	0.000005 (0.000006)	0.8751 (0.9183)
		0–30 min		0–60 min		0–60 min	
4	Fe	0.0025 (0.0023)	0.8868 (0.9056)	0.0000000009 (0.000000001)	0.9127 (0.9474)	0.000000001 (0.000000001)	0.9081 (0.9433)

Note: Data presentation format: kinetic constant *k*_4_ calculated based on the theory-based linear correlation form y = a × x (additional information for comparison is given in brackets—kinetic constant *k*_4_ calculated based on alternative correlation form y = a × x + b with a higher R^2^).

## Data Availability

Data will be made available on request.
